# Understanding the Impact of Deep Learning Model Parameters on Breast Cancer Histopathological Classification Using ANOVA

**DOI:** 10.3390/cancers17091425

**Published:** 2025-04-24

**Authors:** Nerea Hernandez, Francisco Carrillo-Perez, Francisco M. Ortuño, Ignacio Rojas, Olga Valenzuela

**Affiliations:** Department of Computer Engineering, Automation and Robotics, University of Granada, 18071 Granada, Spain

**Keywords:** ANOVA, deep learning, breast cancer subtyping, classification, histologic imaging

## Abstract

Artificial intelligence (AI) has the potential to enhance clinical practice, particularly in the early and accurate diagnosis of diseases like breast cancer. However, for AI models to be effective in medical settings, they must not only be accurate but also interpretable and reliable. This study aims to analyze how variations in different model parameters affect the performance of a weakly supervised deep learning model used for breast cancer detection. Methods: In this work, we apply Analysis of Variance (ANOVA) to investigate how changes in different parameters impact the performance of the deep learning model. The model is built using attention mechanisms, which both perform classification and identify the most relevant regions in medical images, improving the interpretability of the model. ANOVA is used to determine the significance of each parameter in influencing the model’s outcome, offering insights into the specific factors that drive its decision-making. Results: Our analysis reveals that certain parameters significantly affect the model’s performance, with some configurations showing higher sensitivity and specificity than others. By using ANOVA, we identify the key factors that influence the model’s ability to classify images correctly. This approach allows for a deeper understanding of how the model works and highlights areas where improvements can be made to enhance its reliability in clinical practice. Conclusions: The study demonstrates that applying ANOVA to deep learning models in medical applications provides valuable insights into the parameters that influence performance. This analysis helps make AI models more interpretable and trustworthy, which is crucial for their adoption in real-world medical environments like breast cancer detection. Understanding these factors enables the development of more transparent and efficient AI tools for clinical use.

## Simple Summary

Artificial intelligence is becoming an important tool in healthcare, helping doctors detect diseases like breast cancer at early stages. However, for AI to be truly useful, clinicians need to understand how these systems make decisions. In this study, we use a statistical method called Analysis of Variance (ANOVA) to explore how different parameter choices influence the performance of an AI model for breast cancer detection. Beyond classifying images, the model highlights which image regions are most relevant for decision-making. By identifying key factors affecting its behavior, our work contributes to improving the transparency and trust in AI tools in clinical practice.

## 1. Introduction

Breast cancer is the most prevalent type of cancer among women and the leading cause of cancer-related mortality worldwide. According to the World Cancer Statistics (GLOBOCAN) report [1], an estimated 2.3 million new cases would be diagnosed in 2022, representing 23.8% of all new cancer cases in women. Additionally, this report predicted approximately 670,000 deaths, representing 15.4% of cancer-related deaths among female patients. It also accounts for 11.5% of all new cancer cases and 6.8% of all cancer-related deaths across both sexes. These figures underscore the importance of early and accurate diagnosis.

Histologic image analysis is a fundamental aspect of breast lesion identification and characterization. A histologic image is a visual representation of a tissue or biopsy specimen observed under a microscope after preparation and staining with agents such as hematoxylin and eosin (H&E) [2,3]. This process enables the differentiation between distinct cellular and tissue structures, which have been linked to the morphology and spatial organization of these elements and their association with cancer subtypes, grades, and prognosis [2]. Therefore, the development of accurate and efficient methods of histological image analysis, such as those based on machine learning techniques, plays a crucial role in the early detection and diagnosis of breast lesions. These methods enable timely medical interventions that can save lives. This is made possible by the digitization of these images through the use of advanced scanners in the field of digital histopathology. These capture a complete tissue slide at very high resolution in a single digital file called a whole slide image (WSI) [4,5]. It is common to save these images at different magnification levels creating a pyramidal image to perform a detailed analysis of the distinct structures.

Advances in machine learning and computer vision techniques have provided a solution to the challenges of manually analyzing these large, high-resolution images. These challenges include the diverse patterns among subtypes, variations between observers, and the considerable time needed for analysis. These techniques enable the automated classification of images based on lesion subtypes using computational models. These scans can contain millions of pixels and take up several gigabytes of storage space, which makes it challenging to store, process, and analyze them using traditional methods [6,7]. The emergence of Deep Learning (DL) has transformed the field, making it easier to handle these large amounts of data and allowing for the identification of intricate patterns and important features necessary for precise classification [8,9,10,11]. More specifically, Convolutional Neural Networks (CNNs) have revolutionized the way we automatically identify histopathological features associated with various diseases, making the process more efficient and accurate [12,13]. However, building accurate CNN models often demands extensive, costly, and time-consuming creation of large labeled datasets. This can be particularly challenging in histopathology, where obtaining precise local annotations from pathologists is difficult.

This challenge has increasingly led to the adoption of Weakly Supervised Learning (WSL) approaches within the DL framework [14,15,16]. In WSL, the image annotations are global labels, and the goal of the model is to locate regions within the image that are most relevant for classification. Although WSL provides a more efficient method for training DL models, it often requires optimization of various hyperparameters, such as layer configurations and learning rates, to achieve optimal performance. This optimization process usually relies on a trial-and-error approach that may not fully exploit the potential of the model. Therefore, it is critical to complement parameter tuning with rigorous statistical analysis to determine which factors are truly responsible for influencing model performance. Statistical analysis provides a robust framework for selecting the most effective hyperparameters and model architectures to ensure reliable and interpretable results in histological image analysis.

Recent advances in weakly supervised learning (WSL) have changed how we analyze histological images with deep learning. They help solve problems with older methods for segmentation and classification. One key approach is Multiple Instance Learning (MIL) [14,15,17,18,19,20,21,22]. It lets us work with whole slide images (WSIs) without needing detailed labels for every small area. This saves a lot of manual work. MIL uses labels from the whole WSI to make predictions about specific spots, which makes diagnostics faster and more accurate [19,23,24,25]. It sits between segmentation and classification and helps clinicians understand large histological images.

A notable example of weakly supervised learning in histopathology is the CHOWDER model, based in WELDON framework [26] and uses one-dimensional convolutions and a MinMax strategy to find important areas in images [27]. This approach demonstrates strong performance even without detailed local labels. However, CHOWDER was originally designed for binary classification, limiting its applicability to more complex multiclass histopathology tasks.

In this study, we explore the internal mechanisms of a weakly supervised learning (WSL) model to optimize disease detection in whole slide images (WSIs) using only slide-level labels. Our goal goes beyond simply maximizing classification accuracy; we prioritize ensuring that the model is interpretable, allowing physicians to easily understand and trust its predictions. This is particularly crucial in cancer diagnosis, where clear and reliable explanations are essential for effective integration into clinical practice.

To enhance CHOWDER’s capabilities, we introduce several key modifications: increasing the number of convolutional layers, adjusting the architecture of fully connected layers, and extending its functionality to support multiclass classification. These improvements enable the model to distinguish between different subtypes of breast cancer. However, these architectural changes introduce a larger number of hyperparameters, making the optimization process significantly more complex.

To address this challenge, we employ Analysis of Variance (ANOVA) [28] as an alternative to conventional hyperparameter tuning methods such as Grid Search or Random Search. Unlike traditional techniques, which often require extensive computational resources and provide limited insights into individual parameter contributions, ANOVA offers a statistically rigorous approach to identifying the parameters that significantly impact model performance. By quantifying these effects, we transform hyperparameter tuning from a trial-and-error process into a data-driven optimization strategy.

## 2. Materials and Methodology

### 2.1. Data Resource

**Figure 1:**
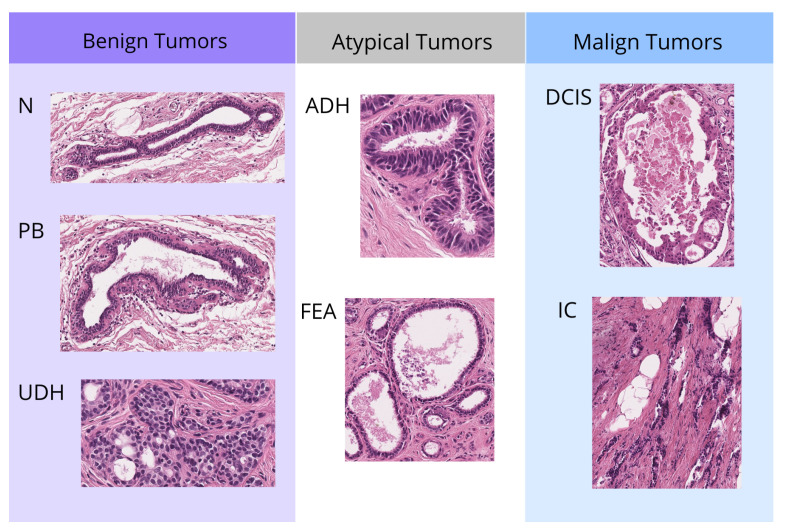
Different subtypes of lesions included in BRACS. Benign lesions include Normal tissue (N), Pathologically Benign (PB), and Usual Ductal Hyperplasia (UDH). Atypical lesions include Atypical Ductal Hyperplasia (ADH) and Flat Epithelial Atypia (FEA). Malignant lesions include Ductal Carcinoma In Situ (DCIS) and Invasive Carcinoma (IC). Representative regions of interest from full tissue slides, shown at 40× magnification, to illustrate differences between lesion classes (benign, atypical, and malignant) based on histological diagnosis.

The BReAst Carcinoma Subtyping (BRACS) dataset [29] is the result of a collaboration between the IRCCS National Cancer Institute of Naples—Pascale Foundation, the Institute for High Performance Computing and Networking (ICAR) of the National Research Council (CNR), and IBM Research (Zurich, Switzerland). As shown in Figure 1, the dataset consists of three types of lesions: benign, malignant, and atypical, further divided into seven subtypes of lesions. Benign lesions include normal tissue (N), pathologically benign (PB), and usual ductal hyperplasia (UDH), while atypical lesions comprise atypical ductal hyperplasia (ADH) and flat epithelial atypia (FEA). Malignant lesions are further classified into Ductal Carcinoma In Situ (DCIS) and Invasive Carcinoma (IC). The images shown are representative regions of interest (ROIs) extracted from full tissue slides. While these ROIs are displayed to highlight specific differences between lesion classes, our analysis is conducted using the complete tissue slides. The dataset contains 547 whole-slide images (WSI) from 189 female patients, which were scanned at a resolution of 0.25 µm/pixel and a magnification factor of 40×. Additionally, the images were annotated by three expert pathologists.

The authors suggest dividing the data as follows: for the training set, they used a total of 395 samples, with 203 benign, 52 atypical, and 140 malignant cases; for the validation set, they used 65 samples, including 30 benign, 14 atypical, and 21 malignant cases; and for the test set, they used a total of 87 samples, consisting of 32 benign, 23 atypical, and 32 malignant cases.

This dataset is particularly interesting because in the case of breast cancer, it’s crucial to differentiate not only between malignant and benign lesions but also atypical ones. Although initially noncancerous, atypical lesions may become malignant in the future.

### 2.2. Model

**Figure 2:**
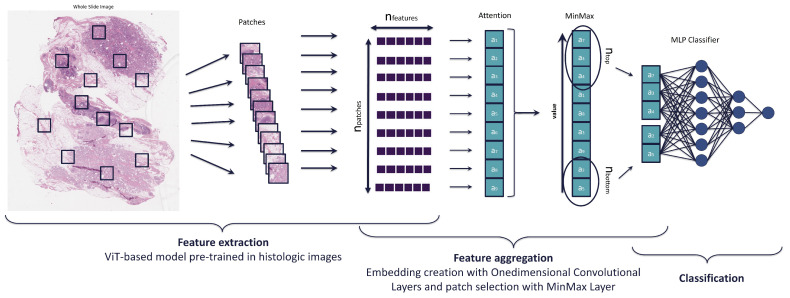
Histological Image Classification Process: Tile extraction by mask to separate tissue from background, ensuring quality; feature extraction with pre-trained iBOT ViT-Base model, obtaining 768-dimensional vectors; one-dimensional convolutional layers for embedding and CAM generation; MinMax layer for selection of patches with positive and negative evidence; and MLP classifier for final classification.

Our approach, shown in Figure 2, is based on the WELDON model, with adaptations and improvements proposed in CHOWDER, which was designed specifically for histopathological image analysis. Firstly, features are extracted from the input data using a pre-trained model. Both WELDON and CHOWDER employ features extracted from ResNet-50, a CNN pre-trained model in ImageNet [30]. In our study, we employ the pre-trained model based on ViT (Vision Transformer), Phikon [31], developed by the same authors as CHOWDER and pre-trained in the same domain, histological images. The model generates a vector of 768 features for each patch or instance.

In this process, we will compute a score for each patch rather than for each pixel, which is more appropriate for histological image classification. To achieve this, we will perform a set of one-dimensional embeddings for these features. For each class, an embedding vector will be constructed, with the number of patches corresponding to the embedding’s length. Each embedding will include an attention score for each of the patches. In our case, these embeddings are obtained by applying five consecutive one-dimensional convolutional layers, with steps applied along the patch index axis for each layer. This one-dimensional convolution resembles a fully connected layer with shared weights between patches. In contrast to the single convolution required by the CHOWDER model, which is suitable for a single-class problem, in our multiclass problem, we require a larger number of consecutive convolutions to obtain good results.

**Figure 3:**
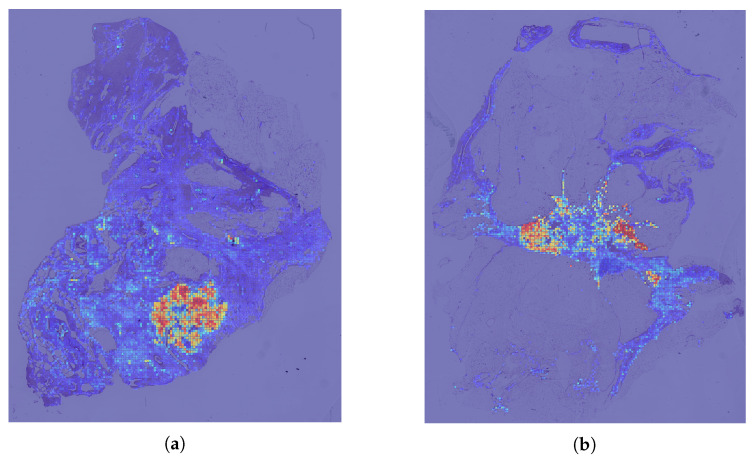
Class Activation Maps (CAMs) for the ‘Malignant’ Class Based on Patch-Level Attention Weights in Two BRACS Samples (**a**,**b**). Red Indicate Higher Attention, Blue Patches Indicate Lower Attention.

Figure 3 illustrates an example of how these attention scores can be used to detect relevant regions in the image. The maps illustrate attention-based activations for two malignant cases. Red areas indicate high similarity to the malignant class, while blue areas indicate low similarity. The highlighted regions suggest that the model’s focus is not random but aligned with meaningful histopathological structures.

Subsequently, a MinMax layer is employed in the output of a one-dimensional convolution layer. This acts as a procedure for selecting patches, whereby embedding values are sorted. For classification, only *n + m* patches per image are utilized, corresponding to the *n* highest and *m* lowest attention scores. It is crucial to highlight that this layer is designed to extract both the most salient instances and negative evidence. Negative evidence is defined as a region that best supports the absence of a given class. During the training phase, the backpropagation algorithm runs through only the selected patches, both positive and negative evidence. We want to check whether this negative evidence provides relevant information. Moreover, in our case, we have not limited ourselves to testing only configurations where *n* and *m* are equal, we wanted to test different configurations to perform a more exhaustive analysis.

As in CHOWDER model, we apply MLP as the final classifier following the MinMax layer. The model in question employs two layers with 200 and 100 neurons, respectively. Tests were conducted on the number of layers for an MLP classifier after selecting the top instances and negative evidence, as suggested in the CHOWDER model. A statistical analysis was then performed to determine the impact of varying the number of layers from one to three.

### 2.3. Statistic Analysis

Analysis of variance (ANOVA) is a powerful statistical tool used to determine if there are significant differences between the means of multiple samples. In the context of deep learning models, ANOVA can be used to analyze the impact on model performance of varying different parameters, which can be measured by accuracy or run time. The goal of this study is to identify which factors affect the performance of our histopathological image classification model to find the best configuration. To achieve this, we have employed the following methodology:

#### 2.3.1. Factor and Level Definitions

In order to proceed, it is necessary to select the parameters of the deep learning model to be studied, called factors in the analysis, and to define different levels for each of them. In the current context, special attention is focused on the following factors:

- Weight decay: regularization during training that penalizes large weights in the model.
Weight Decay (Wd)Level0.10.0
- Layers: the number of layers in the MLP classifier.
Hidden Layers (Ly)LevelNeurons3256, 128, 642128, 64164
- Dropout: percentage of neurons randomly deactivated in these layers to avoid overfitting. Initially, the dropout rates of the first, second, and third neural network layers were included as independent factors in the analysis. However, statistical evaluation revealed that their effects on the outcome variables were not significantly different, indicating analogous behavior across the three layers. To reduce model complexity and avoid redundancy, only the dropout rate of the first layer was retained as a representative factor in the final analysis.
Dropout (Dp)Level0.20.50.8
- Number of top instances: is the number of top patches that are finally used for classification. We divide the images into small fragments or patches of a certain size and train the models with these. This particular model is able to identify the most discriminative or distinctive patches between classes and only uses a certain number for the classification of the whole image.
N Top (Nt)Level5102040
- Number of bottom instances: is the number of bottom patches that are finally used for classification. After the MinMax layer, the patches at the bottom will have the lowest class activation score. These patches will represent what we call “negative evidence”. This term refers to the inclusion of information that indicates the absence of a specific class in an instance or region, as opposed to only considering the presence of positive classes.
N Bottom (Nb)Level051020

#### 2.3.2. Outcome Selection

Once the factors have been defined, it is necessary to choose the metrics or outcomes that will be used to measure the performance of the model in different terms. ANOVA analysis will be performed on each of these metrics, to observe whether there are statistically significant differences between the mean of the results of the tests performed for each of the levels of each factor or whether, on the contrary, it can be assumed that their population means do not differ. In our case, the results chosen to analyze the performance of the model are the following:

- F1-score: is the harmonic mean of precision and recall. Precision measures the proportion of true positives among all instances that the model has labeled as positive. Recall measures the proportion of true positives among all instances that are actually positive. F1-score is a useful metric when a balance between these two aspects is desired and is especially valuable in scenarios with unbalanced classes. Accuracy can be high even if the model does not detect minority classes well. Therefore, the F1 score will help to better assess how the model is performing in those less frequent classes.
- AUC-ROC (“Area Under the Curve” of the “Receiver Operating Characteristic”): in a multiclass classification problem, the One-vs-Rest (OvR) technique evaluates the model’s performance for each class individually. This technique generates an ROC curve for each class, treating it as the positive class and grouping the other classes as negative. The AUC value, which is the area under this curve, measures the model’s ability to distinguish between classes. An AUC value close to 1 indicates excellent performance, while a value close to 0.5 suggests performance similar to chance. The average of the AUC values across all classes provides an overall measure of the model’s performance in multiclass classification.
- Execution Time: measures the total time the model takes to perform the training. It indicates the efficiency of the model in terms of computational resources and time.

Performing an ANOVA analysis for each of these metrics will allow us to understand how different levels of dropout affect not only the model’s accuracy and discrimination ability but also its time efficiency.

#### 2.3.3. Model Training and Data Collection

We trained the model for all combinations of the different parameters. For each run, we store the results in such a way that a tabular dataset will be generated in which each row will correspond to a test, the first columns will store each of the hyperparameters specific to that test, and the following columns will store the results of each of the metrics.

#### 2.3.4. Assumption Validation and Outlier Detection

In the context of ANOVA, the evaluation of the assumptions of normality and homoscedasticity is essential for the validity of the results. Normality can be assessed using graphical methods such as Q-Q plots or statistical tests such as the Shapiro-Wilk test. These methods can be used to determine whether the residuals of the model follow a normal distribution. In addition, the assumption of homoscedasticity, which states that the variance between groups should be equal, can be assessed using residual plots or the Levene test. Confirmation of these assumptions is crucial, as violations of them can lead to inaccurate interpretations and conclusions.

To ensure the validity of ANOVA we implemented a robust outlier detection strategy focused on the primary response variables: AUC and F1 score. Outliers can distort measures of central tendency and variability, potentially biasing model estimates and inferential conclusions.

We first computed studentized residuals for each observation, both with and without the observation included in the calculation of the mean and standard deviation. These residuals, expressed in units of standard deviation, are particularly useful for identifying influential points, as they account for the observation’s leverage. Studentized values are calculated from:

(1) $$z_{i}=\frac{x_{i}-\bar{x}}{s}$$

To increase robustness against deviations from normality, we also calculated modified Z-scores using the median absolute deviation (MAD)—a scale estimator less sensitive to extreme values than the standard deviation. This complementary method allowed for the detection of both conventional and masked outliers.

(2) $$M_{i}=\frac{0.6745(x_{i}-\tilde{x})}{MAD}$$

Additionally, we applied Grubbs’ test [32], which formally tests for a single outlier in a univariate normal distribution. The null hypothesis assumes all data points are drawn from the same normal population; a significant *p*-value (*p* < 0.05) indicates that the most extreme value deviates significantly from this distribution. The test statistic was calculated based on the largest studentized residual (without deletion), using the following formula:

(3) $$T=\sqrt{\frac{n\left(n-2\right)t_{\max}^{2}}{{\left(n-1\right)}^{2}+nt_{\max}^{2}}}$$

where $t_{\max}$ is the maximum absolute studentized value, and *n* is the sample size. An approximate two-sided *p*-value was derived from the Student’s t-distribution with n−2 degrees of freedom and multiplied by 2n, following standard practice. A small *p*-value leads to the conclusion that the most extreme point is indeed an outlier.

#### 2.3.5. ANOVA Analysis

ANOVA is based on comparing between-group variability (different parameter values) with within-group variability (replicates for the same parameter value) [28].

- **Null hypothesis ($H_{0}$)**: The means of the accuracies for the different parameter values are equal.
- **Alternative Hypothesis ($H_{1}$)**: At least one of the means of the accuracies is different.

We calculate the F statistic that compares between-group variability with within-group variability. If the F value is significantly large, the null hypothesis is rejected. If the null hypothesis is rejected, it is concluded that the variation of the parameter has a significant impact on the metric of the model. Otherwise, it cannot be concluded that the parameter has a significant impact.

When the ANOVA confirms the existence of significant differences between groups, it indicates that at least one group differs from the others. However, it does not indicate which group is different. To analyze the pattern of difference between means, ANOVA is usually followed by specific comparisons, such as multiple range tests. Multiple range tests allow multiple comparisons between group means to determine which groups are significantly different from each other. In this study, Fisher’s Least Significant Difference (LSD) procedure [28] was used to distinguish between means. Using this method, there is a 5% risk that each pair of means is significantly different when the true difference equals zero. The main idea of the LSD is to calculate the smallest significant difference between two means as if these means had been the only means to be compared and to declare any difference greater than the LSD as significant.

## 3. Results and Discussion

### 3.1. Model Assumption Validation

#### 3.1.1. Outlier Detection

To ensure the robustness of our statistical analysis and adherence to key ANOVA assumptions (normality and homoscedasticity), we applied a rigorous outlier detection strategy focused on the primary performance metrics: AUC and F1 score.

For AUC, values below 0.65 were identified as outliers. These values are considered insufficient to indicate acceptable model discrimination ability, especially in classification contexts where a random classifier achieves an AUC of 0.5. Statistically, these low values were flagged using studentized residuals, modified Z-scores, and Grubbs’ test, all indicating significant deviation from the expected distribution. Their inclusion distorted residual distribution and variance homogeneity.

For F1, we adopted a similar approach. Given the multi-class nature of the task (three classes), F1 values below 0.45 were deemed indicative of imbalanced performance between precision and recall in at least one class. These cases were also flagged by the same statistical methods and confirmed through residual and boxplot visualizations. Including such underperforming scores would compromise the representativeness of the sample and affect the integrity of model evaluation.

**Figure 4:**
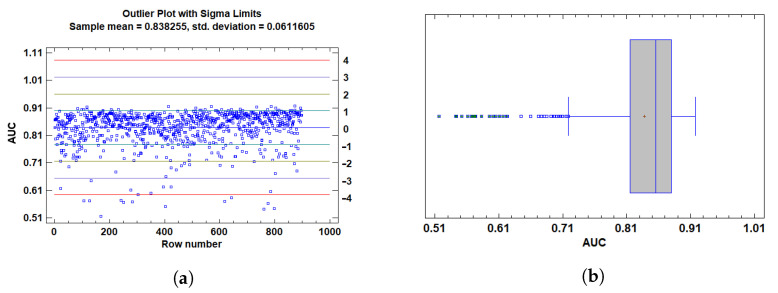
Plots for AUC-ROC outlier detection. (**a**) Outlier plot scores with sigma limits. Each point represents a sample. Horizontal lines indicate the mean (blue) and ±1σ (cyan), ±2σ (green), ±3σ (orange), and ±4σ (red) standard deviation boundaries. Values beyond ±3σ are considered potential outliers. (**b**) Box-and-whisker plot illustrating the interquartile range, median, and whiskers. Outliers and far outliers (beyond 1.5× and 3× the IQR, respectively) are marked with green symbols.

**Figure 5:**
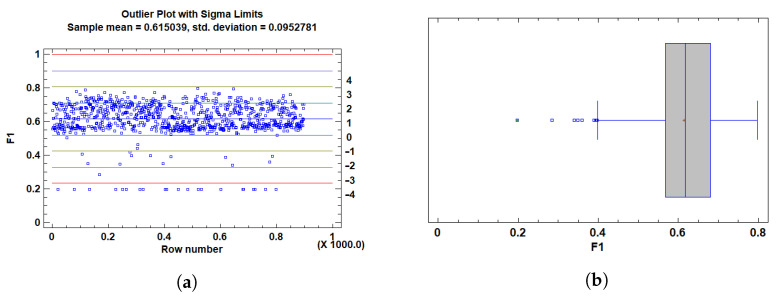
Plots for F1 score outlier detection. (**a**) Outlier plot scores with sigma limits. Each point represents a sample. Horizontal lines indicate the mean (blue) and ±1σ (cyan), ±2σ (green), ±3σ (orange), and ±4σ (red) standard deviation boundaries. Values beyond ±3σ are considered potential outliers. (**b**) Box-and-whisker plot illustrating the interquartile range, median, and whiskers. Outliers and far outliers (beyond 1.5× and 3× the IQR, respectively) are marked with green symbols.

To further justify these exclusions, outlier plots showed in Figure 4 and Figure 5 and box plots were employed to visualize the distribution of the data and identify extreme values. The outlier plot (Figure 4a and Figure 5a) demonstrated that values beyond 3 standard deviations (sigma) significantly deviate from the mean, highlighting potential outliers that could skew the analysis. This visual confirmation aligns with the statistical flags raised by the tests (e.g., studentized residuals and Grubbs’ test), reinforcing the decision to exclude these extreme values.

In addition, the box plot (Figure 4b and Figure 5b) revealed outside points—values more than 1.5 times the interquartile range (IQR) from the box’s edges—and far outside points, which are even more extreme. The box plot visually corroborated the presence of these outliers, further supporting their exclusion based on their deviation from the expected distribution and the potential impact on model evaluation.

Accordingly, and based on both statistical evidence and practical considerations regarding model interpretability and classification adequacy, all observations with AUC<0.65 and F1<0.45 were classified as outliers and excluded from downstream analyses. Finally, we are left with a total of 861 tests.

#### 3.1.2. Residual Validation

After excluding outliers, we prepared the response variables—AUC, F1, and Time—for ANOVA by addressing distributional challenges and validating assumptions.

For AUC, a performance metric bounded between 0 and 1, skewness occurs when values cluster at extremes, risking non-normal residuals and heteroscedasticity, which violate ANOVA assumptions. To address this, we applied the logit transformation:

(4) $$LOGIT\left(p\right)=\ln\left(\frac{p}{1-p}\right)$$

where *p* is the AUC value. This transformation maps the [0, 1] range to (−∞,+∞), reducing skewness, enhancing symmetry, and stabilizing variance to better meet normality and homoscedasticity requirements. In contrast, F1 and Time exhibited distributions that satisfied these assumptions without transformation, allowing the use of their raw values.

**Figure 6:**
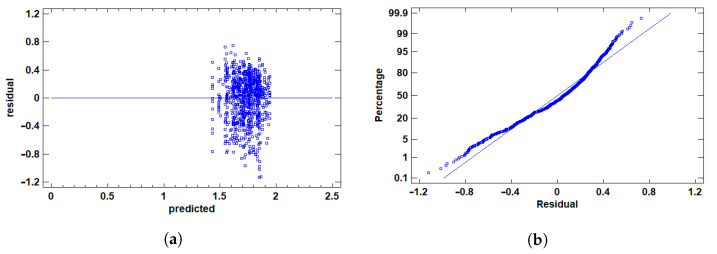
Residual analysis plots for AUC-ROC (transformed). (**a**) Residuals vs. row number. This plot displays the residuals in the order of the data to help identify any patterns or potential influential points. Any systematic pattern may indicate non-random error or the presence of outliers. (**b**) Normal probability plot of residuals. This plot helps assess whether the residuals follow a normal distribution. If so, the points should lie close to the diagonal line.

**Figure 7:**
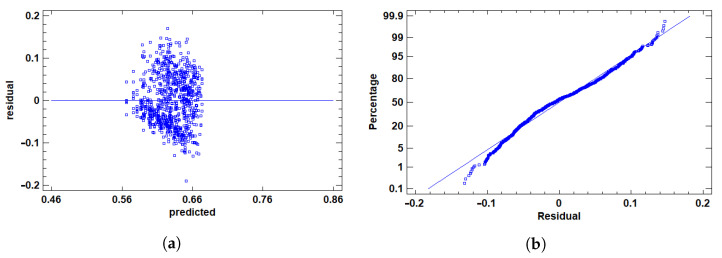
Residual analysis plots for F1 score. (**a**) Residuals vs. row number. This plot displays the residuals in the order of the data to help identify any patterns or potential influential points. Any systematic pattern may indicate non-random error or the presence of outliers. (**b**) Normal probability plot of residuals. This plot helps assess whether the residuals follow a normal distribution. If so, the points should lie close to the diagonal line.

**Figure 8:**
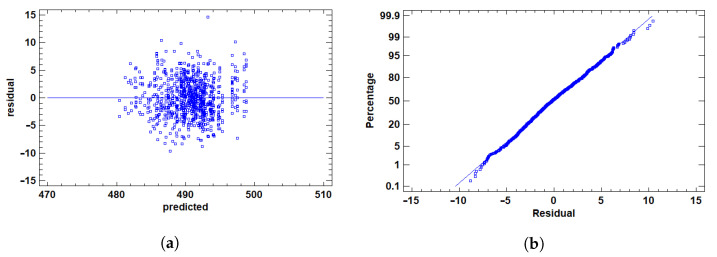
Residual analysis plots for time. (**a**) Residuals vs. row number. This plot displays the residuals in the order of the data to help identify any patterns or potential influential points. Any systematic pattern may indicate non-random error or the presence of outliers. (**b**) Normal probability plot of residuals. This plot helps assess whether the residuals follow a normal distribution. If so, the points should lie close to the diagonal line.

We then graphically validated residuals for all variables using two plots, shown in Figure 6, Figure 7 and Figure 8.

The residuals vs. predicted values plots in Figure 6a, Figure 7a and Figure 8a evaluated homoscedasticity, requiring constant residual variance across levels of the independent variable. Ideally, residuals scatter randomly around zero without patterns. The point cloud was centered at zero, showing no fan shape, structure, or correlation with predicted values for any variable. This confirmed consistent variance, satisfying the homoscedasticity assumption.

The Q-Q plot assessed normality by comparing residual distributions to a normal distribution, as shown in Figure 6b, Figure 7b and Figure 8b. Ideally, points align along a straight line, indicating normality—a key ANOVA assumption. Most points closely followed the line for AUC (post-transformation), F1, and Time, suggesting residuals approximated normality. Slight curvature at the tails was observed but not pronounced, indicating minor deviations unlikely to affect analysis validity.

This approach—transforming AUC while retaining raw F1 and Time values, followed by rigorous graphical validation—ensured all variables met ANOVA assumptions, supporting reliable and accurate results.

### 3.2. Analysis of Variance for F1 Score

The ANOVA table (Table 1) breaks down the variability of F1 based on different factors, with the *p*-values indicating the statistical significance of each factor.

Four *p*-values are less than 0.05, suggesting that these factors have a significant impact on F1 with 95% confidence. The Type III sum of squares was used to measure the contribution of each factor by isolating their effects from the others in the analysis. Specifically, factors the number of layers (Ly), the number of top and bottom instances (Nt and Nb), and the dropout (Dp) were found to have statistically significant effects on F1. Below, we will use a multiple comparison procedure to analyze the data and identify significant differences between the means for each of these factors.

On the other hand, we can also observe that the applied weight decay values do not show considerable variations in the F1 metric, suggesting that the regularization imposed by this parameter does not effectively influence the model’s ability to distinguish between positive and negative classes. Similarly, the number of patches used to represent negative evidence also does not seem to have a noticeable impact on the performance of the model, as measured through the F1 metric. This may indicate that the model is robust to variations in these parameters, or that these factors are not capturing features relevant to classification.

Table 2 reports the Least Squares Means for the F1-score across all tested factor levels, along with their corresponding standard errors and 95% confidence intervals. These values provide a clearer view of how each configuration influences classification performance, complementing the results observed in the ANOVA.

#### 3.2.1. Multiple Range Tests for F1 by Ly

**Figure 9:**
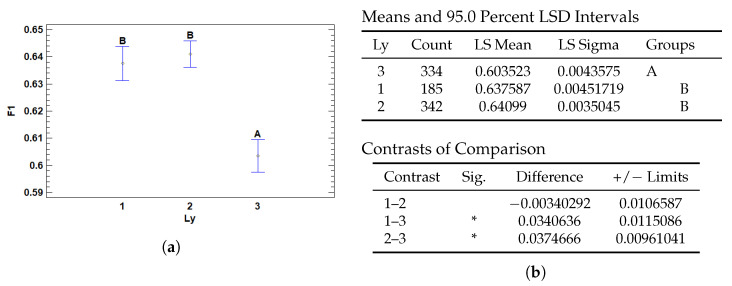
Multiple Range Tests for F1-score by number of hidden layers (Ly). (**a**) Means plot with 95% LSD intervals. Points show group means with confidence intervals; shared letters indicate non-significant differences. (**b**) LSD multiple range test: least squares means and groupings (**top**), and significant contrasts (**bottom**). Asterisks (*) denote statistically significant differences.

The number of hidden layers (*Ly*) in the model significantly affects performance as measured by the F1 score. As shown in Figure 9b, configurations with only one or two hidden layers yield significantly higher F1 scores than those with three layers. Specifically, the model with three layers performs substantially worse, with statistically significant differences observed in the contrasts between *Ly* = 3 and both *Ly* = 1 and *Ly* = 2 (*p* < 0.05). However, no significant difference is observed between the models with one and two hidden layers, indicating similar effectiveness at those depths.

These results suggest that adding more layers beyond a certain point may hinder rather than help performance in this context. While deeper architectures can potentially capture more complex patterns, they also increase model complexity and the risk of overfitting or optimization difficulties—especially in limited data scenarios. The performance drop at three layers may reflect such challenges, including vanishing gradients, noise amplification, or ineffective gradient propagation.

Conversely, shallower architectures (with one or two layers) may offer a better balance between expressiveness and generalizability, especially when the classification task involves subtle but well-defined patterns—as is often the case in histopathology. Figure 9a illustrates this trend, highlighting the performance decline associated with deeper models.

#### 3.2.2. Multiple Range Tests for F1 by Nt

**Figure 10:**
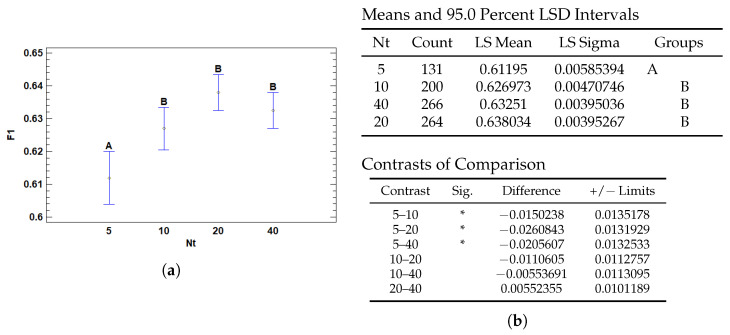
Multiple Range Tests for F1-score by number of Top Patches (Nt). (**a**) Means plot with 95% LSD intervals; points show group means with confidence intervals. Letters indicate non-significant groupings. (**b**) LSD test results: least squares means and groupings (**top**), significant contrasts (**bottom**). Asterisks (*) denote statistically significant differences.

The number of positive instances (*Nt*)—corresponding to patches containing diagnostic features such as tumor tissue—also significantly affects model performance in terms of the F1 score. According to the results in Figure 10b, using only five positive patches per slide leads to significantly lower F1 scores compared to configurations with 10, 20, or 40 positive instances. These differences are statistically significant based on Fisher’s LSD post hoc comparisons, particularly between *Nt* = 5 and all other levels (*p* < 0.05). However, no significant differences were found among 10, 20, and 40 positives, suggesting a performance plateau beyond a minimal threshold.

This finding aligns with the hypothesis that a sufficient amount of positive diagnostic evidence is critical for robust model learning. With only a few positive examples, the model may lack the representational diversity needed to capture the complexity of pathological features, resulting in underfitting and reduced discriminative ability. In contrast, increasing the number of positive patches allows for richer exposure to relevant patterns and morphological variability, which likely contributes to more reliable classification and generalization.

Interestingly, the absence of performance gains between 10 and 40 positive instances suggests that, beyond a certain point, additional positive evidence yields diminishing returns. This plateau may reflect the model’s saturation in learning the core diagnostic signals, reinforcing the importance of balancing data quantity with informativeness. Figure 10a illustrates this trend, showing a marked improvement when increasing from 5 to 10 patches, followed by a stabilization of performance across higher values of *Nt*.

#### 3.2.3. Multiple Range Tests for F1 by Nb

**Figure 11:**
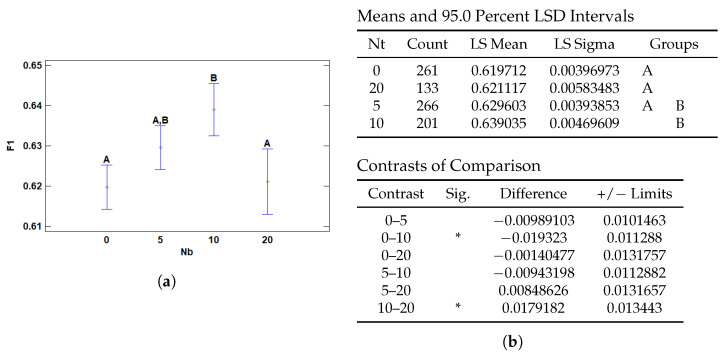
Multiple Range Tests for F1-score by number of Bottom Patches (Nt). (**a**) Means plot with 95% LSD intervals. Letters indicate homogeneous groups. (**b**) LSD test results for Nb: least squares means and standard deviations, followed by significant contrasts. Asterisks (*) indicate statistical significance.

The number of negative instances (*Nb*)—referring to regions that do not contain diagnostic features such as healthy tissue or non-tumoral inflammation—also has a statistically significant effect on F1 score performance. As shown in Figure 11a, there is a non-linear relationship between the amount of negative evidence and model performance. Too few or too many negative patches appear suboptimal, suggesting that a balanced inclusion of negative instances contributes to more robust generalization and enhances diagnostic discrimination in complex histological settings. According to the pairwise comparisons in Figure 11b, configurations with 10 negative instances significantly outperform those with 0 or 20 negative instances, as shown by the contrast results. No significant differences were observed between 0 and 5 or between 5 and 20, although a performance trend is evident.

This result underscores the relevance of incorporating negative evidence during training. The presence of a moderate number of non-diagnostic patches (e.g., *Nb* = 10) appears to improve the model’s ability to contrast relevant and irrelevant regions, akin to how a pathologist reasons by exclusion. This approach is aligned with attention-based models like WELDON, which leverage both high-activation (positive) and low-activation (negative) patches to contextualize decisions.

#### 3.2.4. Multiple Range Tests for F1 by Dp

**Figure 12:**
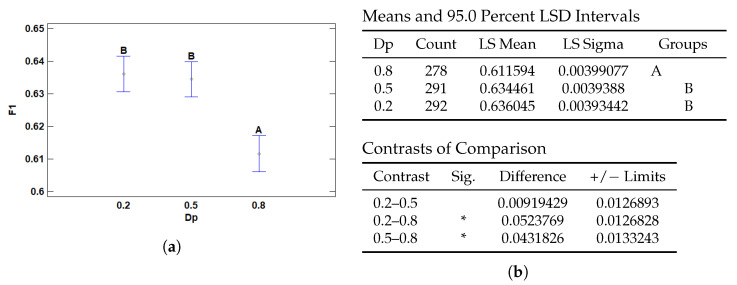
Multiple Range Tests for F1-score by dropout rate (Dp). (**a**) Means plot with 95% LSD intervals. Letters represent homogeneous groups. (**b**) LSD multiple range test for Dp showing group means and significant pairwise differences (marked with *).

The dropout rate of the first layer also significantly affects the F1 score. As shown in Figure 12b, a dropout value of 0.8 results in significantly lower F1 performance compared to 0.5 and 0.2, as confirmed by the pairwise contrasts. Conversely, no statistically significant difference was found between 0.2 and 0.5, indicating similar performance between these configurations.

The markedly reduced F1 score at a dropout of 0.8 may be attributed to excessive regularization, which can lead to information loss, reduced model capacity, and convergence issues. In contrast, dropout values of 0.2 and 0.5 appear to strike a better balance between regularization and model expressiveness, preserving predictive performance.

As illustrated in Figure 12a, although dropout rates of 0.2 and 0.5 yield comparable results, the overall trend shows a monotonic decrease in F1 score as dropout increases. The performance gap between a dropout rate of 0.8 and the other two levels is more pronounced than that between 0.2 and 0.5.

### 3.3. Analysis of Variance for AUC-ROC

A new ANOVA analysis was then performed for the AUC-ROC metric to see which factors affected the discrimination ability of the model.

As can be seen in the Table 3, the factors affecting this metric are the number of top patches (Nt) and the number of bottom patches (Nb). Compared to the case of the F1-score metric, the number of layers and the dropout rate do not statistically affect the model discriminability.

Table 4 presents the Least Squares Means for the Area Under the ROC Curve (AUC) for each experimental setting. The accompanying standard errors and confidence intervals help to assess the stability and reliability of the model’s discriminative ability under different parameter combinations.

#### 3.3.1. Multiple Range Tests for AUC by Nt

**Figure 13:**
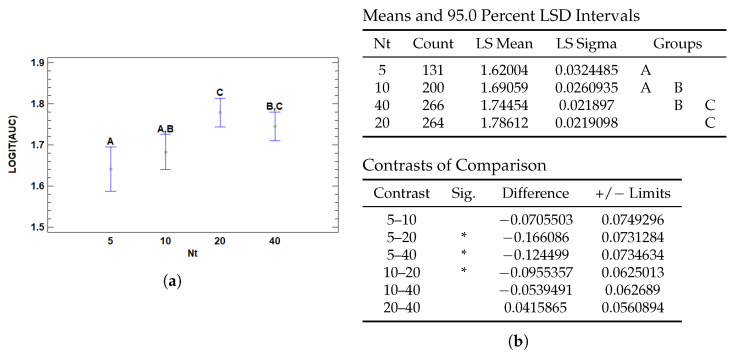
Multiple Range Tests for AUC-ROC by number of Top Patches (Nt). (**a**) LSD means plot with confidence intervals and group letters. (**b**) LSD test for Nt: least squares means and pairwise contrasts with significant differences marked by asterisks.

The number of positive instances (*Nt*) significantly affects model performance in terms of the logit-transformed AUC (Area Under the ROC Curve), as shown in Figure 13. Statistically significant differences were found between several levels of *Nt*, particularly between *Nt* = 5 and both *Nt* = 20 and *Nt* = 40, as well as between *Nt* = 10 and *Nt* = 20. This indicates a marked improvement in AUC performance as the number of positive instances increases from low to moderate levels.

Models trained with only 5 positive instances per slide achieved significantly lower AUC scores compared to those with 20 or 40, suggesting that limited exposure to diagnostically relevant regions impairs the model’s capacity to generalize. With such low signal, the model may struggle to capture the essential patterns needed to distinguish between classes, leading to unreliable performance.

This pattern mirrors what was observed with the F1-score. There, a low number of positive instances (Nt = 5) also led to significantly poorer F1 performance compared to higher counts, particularly Nt = 20 and 40. However, while the F1-score differences between Nt = 10, 20, and 40 were not statistically significant, the AUC metric continued to show a subtle but progressive improvement up to Nt = 20. This suggests that AUC is more sensitive to incremental improvements in the model’s discrimination ability across the full range of predictions, while F1—being threshold-dependent—is more influenced by classification decisions near the decision boundary.

#### 3.3.2. Multiple Range Tests for AUC by Nb

**Figure 14:**
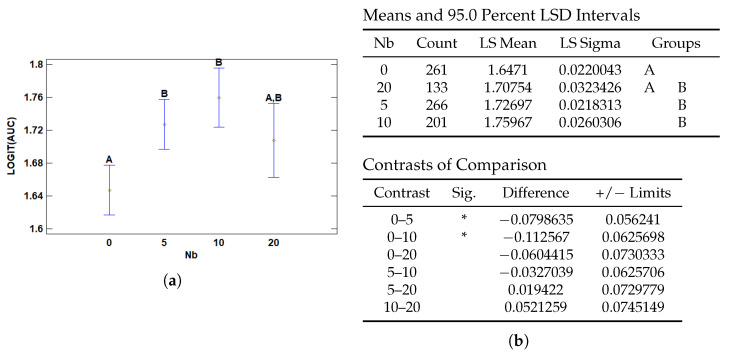
Multiple Range Tests for AUC-ROC by number of Bottom Patches (Nb). (**a**) Means plot with 95% LSD intervals and groupings. (**b**) LSD multiple range test output for Nb, showing group differences and significance levels marked by asterisks.

The number of negative instances per slide (*Nb*) also showed a significant effect on model performance in terms of AUC, as presented in Figure 14. Specifically, models trained with no negative instances (*Nb* = 0) exhibited significantly lower AUC values compared to those trained with 5 or 10 negative instances. This suggests that the absence of negative evidence may limit the model’s ability to learn meaningful contrast between relevant and irrelevant regions.

In the AUC analysis, the most pronounced difference was between *Nb* = 0 and *Nb* = 10, with an estimated logit-AUC difference of −0.113, which was statistically significant. Meanwhile, differences among higher values of *Nb* (5, 10, 20) were not significant, suggesting a plateau effect where once a sufficient amount of negative evidence is present, additional instances do not yield further gains.

This trend aligns closely with the findings in the F1-score analysis, where models trained without negative instances also underperformed significantly compared to those with some level of negative context. However, F1 did not show statistically significant differences among *Nb* = 5, 10, and 20, further highlighting that AUC may be more sensitive to subtle gains in discriminative performance across the prediction spectrum.

### 3.4. Analysis of Variance for T (s)

Finally, a new ANOVA analysis was performed to examine the impact of different factors on the run time. The results of this analysis are presented in Table 5.

Analysis of variance (ANOVA) reveals that several factors have a significant impact on training time (T). Specifically, the weight decay (Wd), the number of hidden layers (Ly) and the number of top and bottom patches (Nt and Nb) show *p*-values less than 0.05, indicating that they have a statistically significant effect on training time at the 95% confidence level.

Table 6 displays the Least Squares Means for execution time, offering insight into the computational efficiency of each tested configuration. The inclusion of confidence intervals allows for a better comparison of the time-related trade-offs identified in the ANOVA results.

#### 3.4.1. Multiple Range Tests for T (s) by Wd

**Figure 15:**
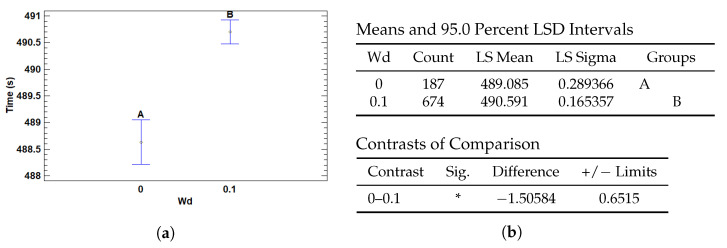
Multiple Range Tests for training time by weight decay (Wd). (**a**) LSD plot of group means and intervals. (**b**) LSD test tables for Wd showing group comparisons and significant differences (*).

The regularization parameter weight decay (Wd) was found to have a statistically significant impact on execution time. As shown in Figure 15, the model configurations with no weight decay (Wd = 0) achieved significantly faster execution times compared to those with a weight decay of 0.1. Specifically, the mean execution time for Wd = 0 was 489.09 s, whereas for Wd = 0.1 it increased to 490.59 s.

The contrast analysis confirms this difference is statistically significant, with a mean difference of approximately 1.51 s and a 95% confidence interval that excludes zero. This suggests that introducing weight decay, while often beneficial for generalization and model regularization, introduces a modest computational overhead.

Although the difference in absolute terms is small, the effect may become relevant in large-scale deployments or hyperparameter sweeps where training efficiency is critical. The additional cost likely stems from more complex gradient updates during optimization when regularization is applied.

#### 3.4.2. Multiple Range Tests for T (s) by Ly

The number of layers in a neural network also affects the execution time. The greater the number of layers, the greater the amount of computation required during forward propagation and backpropagation during training. Each additional layer adds computational complexity, thus increasing the execution time.

**Figure 16:**
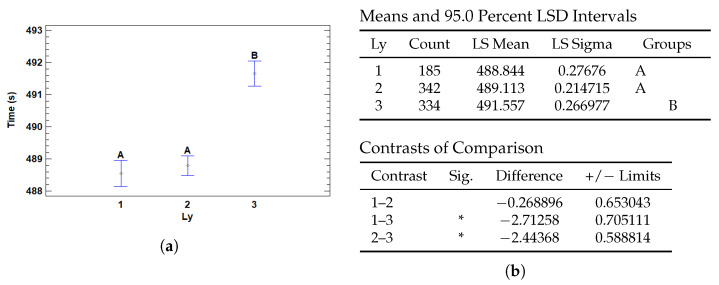
Multiple Range Tests for training time by number of hidden layers (Ly). (**a**) Plot of group means and LSD intervals. (**b**) LSD test results showing significant differences (*) for Ly configurations.

In Figure 16 we can see that the one- and two-layer models have similar and shorter execution times compared to the three-layer model. They have lower complexity and therefore require less processing time. Models with one and two layers do not show significant differences in execution time, which may be due to the similarity in the amount of computations required.

The complexity and number of computations increase with more layers, which explains the significant increase in execution time for the model with three layers.

#### 3.4.3. Multiple Range Tests for T (s) by Nt

The number of patches used for training increases the number of data processed and computational operations, which in turn increases the execution time. Each patch represents an additional portion of the data that must be analyzed and processed by the model, thus increasing the amount of inputs the model needs to handle. Consequently, the additional processing required for each additional patch translates into a significant increase in run time, due to the increased computational load and the time needed to process and train with a larger volume of information.

**Figure 17:**
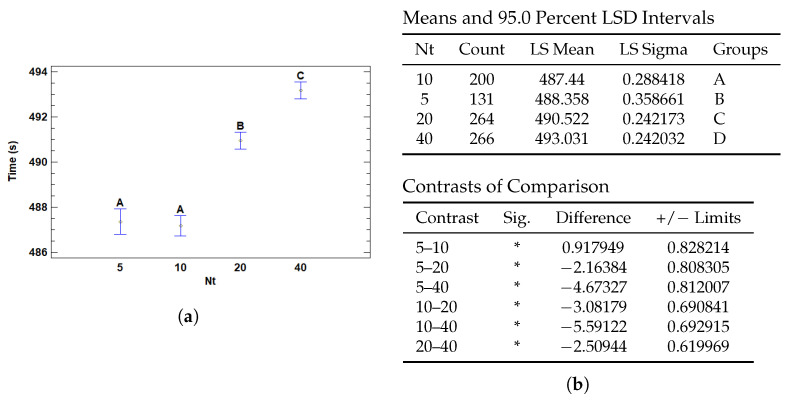
Multiple Range Tests for training time by number of top patches (Nt). (**a**) Means plot with 95% LSD intervals. (**b**) Multiple range test for Nt showing differences between groups, with significant contrasts marked by *.

The results in Figure 17 show that the number of patches used for classification significantly affects the run time. The models with 5 and 10 patches have similar and relatively low run times, with no significant differences between them. However, the models with 20 and 40 patches have significantly longer run times. Specifically, the run time of the 40-patch model is the longest and differs significantly from the others. This increase in run time with more patches suggests that, while increasing the number of patches may improve classification accuracy, it also increases computational complexity, requiring a trade-off between the number of patches and processing time.

#### 3.4.4. Multiple Range Tests for T (s) by Nb

The number of bottom patches as well as the top increases the number of total patches used for training which increases the number of data processed and computational operations, which in turn increases the execution time.

**Figure 18:**
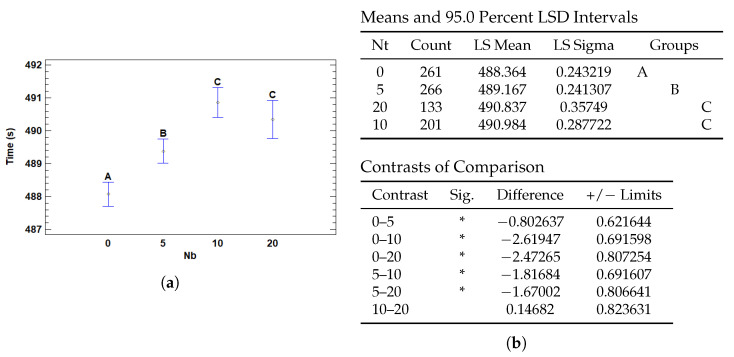
Multiple Range Tests for training time by number of bottom patches (Nb). (**a**) LSD means plot with confidence intervals. (**b**) LSD pairwise test results for Nb, including group means and statistically significant differences (*).

The results in Figure 18 reveal that the use of a higher number of patches significantly influences the execution time, leading to a noticeable increase when 10 or more patches are added. The lowest mean time is observed with 0 patches, and although the time increases slightly with 5 patches, it becomes significantly longer when using 10 and 20 patches. The differences are significant between groups without patches and those with 10 and 20, while there are no significant differences between 5, 10 and 20 patches. This suggests that while adding patches may improve performance, it also increases run time, especially when exceeding 10 patches, highlighting the need to balance model complexity and processing time efficiency.

## 4. Conclusions

The development of AI models for breast cancer diagnosis requires more than achieving high classification accuracy: it requires robustness, interpretability, and alignment with clinical reasoning. In this study, we went beyond traditional trial-and-error hyperparameter fitting and adopted a rigorous statistical framework based on analysis of variance (ANOVA). This allowed us not only to identify the most effective parameter settings, but also to understand the underlying causes of their performance, which parameters actually affect the results.

Our results show that moderate dropout rates (e.g., 0.2–0.5) provide a favorable balance between regularization and learnability versus higher regularization (0.8). The latter makes the model unable to train satisfactorily. In addition, we find that deeper architectures of the final classifier, beyond two or three layers, do not provide greater gains and may even impair performance. Next, we observed that eliminating weight decay significantly reduced training time without affecting accuracy.

As for instance selection, increasing the number of positive patches from 5 to 10 or 20 improved both F1 score and AUC markedly, while gains stagnated beyond that point. The addition of negative evidence-nondiagnostic regions-also produced significant improvements in performance, supporting the idea that context plays a vital role, as in a pathologist’s reasoning. In both cases, the higher the number of instances, the longer the execution time, which supports the use of average terms such as 10–20 positive instances and 5–10 negative instances would be sufficient.

These results reinforce the principles of learning theory, emphasizing the simplicity of the model, and highlight the biological plausibility of focusing on mid-level features such as nuclear morphology and glandular structures. In addition, we ensured the statistical rigor of our findings by verifying ANOVA assumptions, applying logit transformations when necessary, and carefully removing outliers using statistical and technical criteria.

Going forward, this work lays the groundwork for a more principled and interpretable approach to model optimization in computational pathology. Future work will compare with other models, incorporate advanced explainability techniques, and evaluate generalization to larger and more diverse data sets. Ultimately, we believe that combining rigorous statistical analysis with interpretable deep learning architectures can accelerate the safe and effective integration of AI into diagnostic workflows. 

## Figures and Tables

**Table 1:** ANOVA results for F1 Score-Type III Sums of Squares.

Source	Sum of Squares	Df	Mean Square	F-Ratio	*p*-Value
Main Effects					
A: Wd	0.000697502	1	0.000697502	0.20	0.6566
B: Ly	0.224158	2	0.112079	31.76	0.0000
C: Nt	0.054888	3	0.018296	5.19	0.0015
D: Nb	0.0474815	3	0.0158272	4.49	0.0039
E: Dp	0.105687	2	0.0528436	14.98	0.0000
Residual	2.99578	849	0.0035286		
Total	3.47639	860			

**Table 2:** Means Table for F1 by Level.

Factor	Level	Mean	Stnd. Error	Lower Limit	Upper Limit
Grand Mean		0.627367			
Wd	0.0	0.626161	0.00472293	0.616904	0.635418
	0.1	0.628573	0.0026989	0.623283	0.633863
Ly	1	0.637587	0.00451719	0.628734	0.646441
	2	0.64099	0.0035045	0.634121	0.647859
	3	0.603523	0.0043575	0.594983	0.612064
Nt	5	0.61195	0.00585394	0.600476	0.623423
	10	0.626973	0.00470746	0.617747	0.6362
	20	0.638034	0.00395267	0.630287	0.645781
	40	0.63251	0.00395036	0.624768	0.640253
Nb	0	0.619712	0.00396973	0.611932	0.627493
	5	0.629603	0.00393853	0.621884	0.637323
	10	0.639035	0.00469609	0.629831	0.648239
	20	0.621117	0.00583483	0.609681	0.632553
Dp	0.2	0.636045	0.00393442	0.628334	0.643757
	0.5	0.634461	0.0039388	0.626741	0.642181
	0.8	0.611594	0.00399077	0.603773	0.619416

**Table 3:** ANOVA results for LOGIT(AUC-ROC)-Type III Sums of Squares.

Source	Sum of Squares	Df	Mean Square	F-Ratio	*p*-Value
Main Effects					
A: Wd	0.0720822	1	0.0720822	0.66	0.4148
B: Ly	0.417128	2	0.208564	1.92	0.1467
C: Nt	2.40306	3	0.801019	7.39	0.0001
D: Nb	1.54485	3	0.514949	4.75	0.0027
E: Dp	0.145252	2	0.0726261	0.67	0.5120
Residual	92.0456	849	0.108417		
Total	97.7375	860			

**Table 4:** Means Table for LOGIT(AUC-ROC) by Level.

Factor	Level	Mean	Stnd. Error	Lower Limit	Upper Limit
Grand Mean		1.71032			
Wd	0.0	1.69806	0.0261793	1.64675	1.74937
	0.1	1.72258	0.0149601	1.69326	1.7519
Ly	**1**	1.67482	0.0250389	1.62575	1.7239
	2	1.73297	0.0194255	1.6949	1.77105
	3	1.72316	0.0241537	1.67582	1.7705
Nt	5	1.62004	0.0324485	1.55644	1.68363
	10	1.69059	0.0260935	1.63944	1.74173
	20	1.78612	0.0219098	1.74318	1.82907
	40	1.74454	0.021897	1.70162	1.78745
Nb	**0**	1.6471	0.0220043	1.60397	1.69023
	5	1.72697	0.0218313	1.68418	1.76975
	10	1.75967	0.0260306	1.70865	1.81069
	20	1.70754	0.0323426	1.64415	1.77093
Dp	0.2	1.70784	0.0218086	1.66509	1.75058
	0.5	1.72737	0.0218329	1.68458	1.77016
	0.8	1.69576	0.0221209	1.6524	1.73911

**Table 5:** ANOVA results for Time (s)-Type III Sums of Squares.

Source	Sum of Squares	Df	Mean Square	F-Ratio	*p*-Value
Main Effects					
A: Wd	271.83	1	271.83	20.52	0.0000
B: Ly	1087.5	2	543.749	41.05	0.0000
C: Nt	3696.49	3	1232.16	93.02	0.0000
D: Nb	914.478	3	304.826	23.01	0.0000
E: Dp	10.8907	2	5.44534	0.41	0.6630
Residual	11,245.6	849	13.2457		
Total (Corrected)	19,650.1	860			

**Table 6:** Means Table for Time by Level.

Factor	Level	Mean	Stnd. Error	Lower Limit	Upper Limit
Grand Mean		489.838			
Wd	0.0	489.085	0.289366	488.518	489.652
	0.1	490.591	0.165357	490.267	490.915
Ly	1	488.844	0.27676	488.302	489.386
	2	489.113	0.214715	488.692	489.534
	3	491.557	0.266977	491.033	492.08
Nt	5	488.358	0.358661	487.655	489.061
	10	487.44	0.288418	486.875	488.005
	20	490.522	0.242173	490.047	490.996
	40	493.031	0.242032	492.557	493.506
Nb	0	488.364	0.243219	487.887	488.841
	5	489.167	0.241307	488.694	489.64
	10	490.984	0.287722	490.42	491.547
	20	490.837	0.35749	490.136	491.537
Dp	0.2	489.701	0.241055	489.228	490.173
	0.5	489.974	0.241324	489.501	490.447
	0.8	489.839	0.244507	489.359	490.318

## Data Availability

The data presented in this study are available in https://www.bracs.icar.cnr.it/.
